# DEPP1 connects nutrient and oxygen availability to maintenance of muscle mass

**DOI:** 10.64898/2026.09.21.753276

**Published:** 2026-09-27

**Authors:** Shariq Qayyum, Christopher Lavallee, Madhu Singh, Gregory Wyant

## Abstract

Nutrients and oxygen are sensed within the muscle to control growth and disruption of either signal is sufficient to lead to muscle atrophy. While nutrient limitation is sensed via a conserved transcriptional atrophy program (commonly referred to as atrogenes) dictated via the Forkhead box O (FoxO) transcription factors, how low oxygen promotes muscle loss remains unknown. Accordingly, the downstream mechanisms that initiate muscle loss when oxygen and nutrients are limiting are only partly understood. Here, we find Hypoxia Inducible Factor (HIF), the master regulator of our adaptation to low oxygen, is necessary and sufficient to mediate muscle loss under hypoxia in mice. RNA sequencing in skeletal muscle isolated from starved or hypoxic mice identifies Decidual Protein Induced by Progesterone 1 (Depp1), which is induced in skeletal muscle when nutrients or oxygen is limiting via FoxO1 and HIF activation, respectively. Whole body Depp1 loss in mice reduces muscle loss under fasting and hypoxia and skeletal muscle Depp1 overexpression is sufficient to mediate muscle atrophy. Mechanistically, Depp1 localizes to the mitochondria and is necessary to control autophagy activation and mitochondrial degradation in skeletal muscle. Taken together, our studies nominate Depp1 as a new atrogene necessary for muscle loss under multiple atrophy scenarios involving FoxO and HIF.

## Full Text Availability

The license terms selected by the author(s) for this preprint version do not permit archiving in PMC. The full text is available from the preprint server.

